# Stability and Dynamic Aggregation of Bare and Stabilized Zero-Valent Iron Nanoparticles under Variable Solution Chemistry

**DOI:** 10.3390/nano10020192

**Published:** 2020-01-22

**Authors:** Hesham M. Ibrahim, Mohammed Awad, Abdullah S. Al-Farraj, Ali M. Al-Turki

**Affiliations:** 1 Department of Soil Science, College of Food and Agricultural Sciences, King Saud University, P.O. Box 2460, Riyadh 11451, Saudi Arabia; awad.mohammed333@yahoo.com (M.A.); sfarraj@ksu.edu.sa (A.S.A.-F.); aturki@ksu.edu.sa (A.M.A.-T.); 2 Department of Soils and Water, Faculty of Agriculture, Suez Canal University, Ismailia 41522, Egypt

**Keywords:** nanoscale zero-valent iron (nZVI), carboxymethyl cellulose (CMC), dynamic aggregation, pH, ionic strength (IS)

## Abstract

Surface modification of nanoscale zero-valent iron (nZVI) using polymer stabilizers (e.g., sodium carboxymethyl cellulose, CMC) is usually used to minimize aggregation, increase stability, and enhance transport of nZVI. We investigated the stability and dynamic aggregation of bare and CMC–nZVI as affected by variations in pH, ionic strength (IS), and nZVI particle concentration. CMC coating of nZVI resulted in smaller hydrodynamic size and larger zeta potential. The largest hydrodynamic size of nZVI was associated with bare nZVI at high IS (100 mM), pH close to the point of zero charge (PZC, 7.3–7.6), and larger particle concentration (1.0 g L^−1^). The increase in the zeta potential of CMC–nZVI reached one- to four-fold of that for bare nZVI, and was greater at pH values close to PZC, high IS, and larger particle concentration. The stability of CMC–nZVI was increased by 61.8, 93.1, and 57.5% as compared to that of bare nZVI at IS of 1, 50 and 100 mM, respectively. Calculations of Derjaguin, Landau, Verwey and Overbeek (DLVO) interaction energy were in agreement with stability results, and showed the formation of substantial energy barriers at low IS indicating greater nZVI stability. Our results suggest that at IS above 50 mM and nZVI particle concentration larger than 0.1 g L^−1^, the likelihood of nZVI aggregation is high. Nevertheless, CMC polymer stabilizer would enhance the stability and transport of nZVI even under these unfavorable solution chemistry conditions.

## 1. Introduction

Nanoscale zero-valent iron (nZVI) has been widely used for the remediation and detoxification of groundwater and soil resources contaminated by a variety of environmental contaminants, including chlorinated organic solvents, organochlorine pesticides, polychlorinated biphenyls (PCBs), polycyclic aromatic hydrocarbons (PAHs), and metal ions [1,2,3]. Due to their nano-sized dimensions, nZVI have significantly large surface area relative to their volume, and higher reactivity at the reactive surface sites [4], which subsequently enhance its capabilities in contaminant degradation reactions [5]. Despite the higher activity of nZVI, several studies have shown that nZVI particles exhibit strong tendency of agglomeration, rapid sedimentation, and limited mobility in the aquatic environment, which represent a major challenge for environmental applications of nZVI [6,7]. For example, Phenrat et al. [8] reported the formation of micro-sized aggregates of uncoated nZVI in aqueous solutions within 30 min. Therefore, research has focused on modifying the surfaces of nZVI by applying different coating materials to reduce aggregation and settling so that the nZVI nanoparticles remain dispersed in aqueous suspensions for longer times [9]. 

Polymers and surfactants are among the main types of materials used as coatings for nZVI, including starch [6], xanthan gum [10], polyacrylic acid [11], polystyrene sulfonate [12], and carboxymethyl cellulose (CMC) [13]. Among the different coating materials, CMC (a food grade, biodegradable anionic polymer), has received greater attention, and showed great potential to stabilize nZVI particles in many laboratory and field applications [14,15,16]. Stabilization of nZVI using coating materials rely on combined electrosteric stabilization of nZVI. Electrostatic repulsion is achieved by increasing the surface charge to increase the repulsive forces between particles, whereas steric stabilization is attained by the adsorption of long-chain organic molecules, which hinders particle attraction [12,13,17]. 

Prolonged nZVI nanoparticle stability in aqueous suspensions has led to greater mobility in subsurface layers, and consequently enhanced remediation activities. The extent of the stability of coated nZVI nanoparticles is dependent on polymer characteristics, thickness of the polymer layer, and solution chemistry [12]. The amount of polyelectrolyte used in the coating process greatly affects the total surface area of nZVI, directly impacting aggregation of nZVI and its stability in aqueous suspensions [16]. Gordon et al. [18] reported that in the absence of any coating agent almost all of the nZVI particles were settled out. In contrast, CMC-stabilized monometallic nZVI remained stable for a long time and was able to decrease trichloroethane (TCE) concentrations in groundwater by more than 90% [19]. 

The stability of nZVI also depends on solution chemistry (i.e., pH and ionic strength). Variations in the pH of nZVI suspensions affect the type and amount of charges on the surfaces of nZVI. The electrostatic properties of nZVI in aqueous suspensions are controlled by the pH of the suspension, with minimum nZVI stability occurring when pH approaches the point of zero charge (PZC) [20]. The stability of nZVI is increased at larger zeta potential, whereas at lower zeta potential nZVI particles tend to aggregate rapidly [21].

The influence of ionic strength (IS) on the stability and mobility of nZVI in water has been extensively examined [22,23]. In general, increasing IS increases the efficiency of particle-collector attachment and reduces the stability and mobility of nZVI [15]. In the case of hydrophilic polymers or polyelectrolyte surface coatings, it was found that electrostatic stabilization by coating of charged polymers or surfactants is sensitive only at low ionic strength. At high ionic strength, these coating materials are unlikely to be effective [6,13,17]. Jeremy at al. [22] observed a clear trend of increasing particle aggregation with increasing ionic strength. At low ionic strength (e.g., <0.1 M), a larger energy barrier exists between smaller particles that prevents particles from coming closer to each other and hence maintains particle stability. At high ionic strength (e.g., >0.1 M), the energy barrier is eliminated for all particle sizes, which implies that colloidal aggregation is more severe at elevated ionic strength [23].

The stability of nZVI is also affected by the volume fraction of nZVI particles. At larger nZVI concentrations, the range of the interaction forces between particles is closer, and the interaction is dominated by the van der Waals and magnetic attractive forces, which increase aggregation and sedimentation of particles [12]. Research has shown that nZVI aggregation is directly proportional to the concentration of nZVI, especially at larger particle concentration above 0.1 g L^−1^ [24]. 

The overall goal of this research was to assess best synthesizing conditions and solution chemistry to ensure greater nZVI stability in aqueous environments. The specific objectives were to: (1) investigate the effect of CMC surface coating and nZVI particle concentration on the dynamic aggregation of nZVI; (2) evaluate the effect of pH (5–11) and IS (1–100 mM) on the hydrodynamic size, surface charge, and stability of nZVI in aqueous suspensions; and (3) apply the classical Derjaguin, Landau, Verwey and Overbeek (DLVO) theory to qualitatively assess nZVI particle interaction forces, and help to interpret the behavior and fate of nZVI in aqueous suspensions.

## 2. Materials and Methods

### 2.1. Preparation of nZVI

nZVI were synthesized by the reduction of ferric chloride by sodium borohydride [25]. All chemicals used in this study were of analytical grade. FeCl_3_.6H_2_O, NaHCO_3_, NaBH_4_, and Carboxymethyl cellulose (CMC, MW = 90,000) were purchased from Sigma Aldrich. All solutions were prepared in nanopure water, which was purged with N_2_ for 1 h prior to usage. nZVI–CMC were prepared freshly by adding 0.1 M NaBH_4_ aqueous solution drop wise to a 0.1 M FeCl_3_.6H_2_O, and adding 200 mL CMC with 2% concentration at ambient temperature and under atmospheric conditions. The preparation of solutions was carried out as follows: 2.5406 g of FeCl_3_.6H_2_O was dissolved in 24 mL ethanol + 6 mL nanopure water (0.1 M FeCl_3_.6H_2_O). The CMC solution was prepared by dissolving 0.4 g in 200 mL nanopure water; the solution was stirred with a mechanical stir at 750 rpm for 30 min. The sodium borohydride (NaBH_4_, 0.3783 g) powder was dissolved in 100 mL nanopure water (0.1 M NaBH_4_), and was added to the mixture drop wise (1 drop per 2 s). Stirring was continued for another 30 min. The suspension was filtered (Whatman 42) under vacuum, washed with 25 mL ethanol three times, and transported to storage bottle. The preparation of bare nZVI was carried out following the same previous steps without the addition of CMC. 

After the completion of the nZVI preparatory phase, the concentration of the prepared nZVI was determined (~3.0 g L^−1^), and stored as a stock suspension. Two final nZVI concentrations of 0.1 and 1.0 g L^−1^ were prepared in NaHCO_3_ background electrolyte solution. nZVI suspensions were prepared in different IS concentrations of 1, 50, and 100 mM, and the pH of the suspensions was adjusted at 5, 7, 9, and 11 using 0.01 M HCl or 0.01 M NaOH. All suspensions were sonicated for 10 min prior to use.

### 2.2. Characterization of the Prepared nZVI

X-ray diffraction (XRD) was used to analyze the crystalline structure of the prepared nZVI particles. XRD analysis was carried out immediately after preparation and after an aging period of one month. The XRD analysis was conducted with an Altima IV X-ray diffractometer (Rigaku, Austin, TX, USA). Iron nanoparticles were placed in a glass holder and scanned from 20° to 90° at a scanning rate of 2.0° min^−1^. Transmission electron microscopy (TEM–JEOL, JEM1011), (JEOL, Inc., Peabody, MA, USA) was used to characterize the shape of the nZVI particles. The specific surface area of the nZVI particles was determined by the Brunauer, Emmett and Teller (BET) method with nitrogen (N_2_) at 77 K using surface area and microporosity analyzer (ASAP 2020, Micromeritics, Norcross, GA, USA).

The hydrodynamic size of nZVI particles in aqueous suspensions was measured using laser Doppler velocimetery (Zetasizer Nano ZS, Malvern, UK). Zeta potential of nZVI nanoparticles was determined by dynamic light scattering techniques by measuring the electrophoretic mobility for the different particle suspensions using Zetasizer Nano ZS with MPT2 titrator. The point of zero charge (PZC) was determined over the pH range of 2–12. Averaged values for the hydrodynamic size and zeta potential of nZVI were obtained from 10 measurements, and were presented along with standard deviation (±1 SD).

### 2.3. Stability and Dynamic Aggregation of nZVI

Colloidal stability of iron nanoparticles has been defined as the tendency to disperse in solvent against aggregation during specific time period [26]. Dynamic aggregation of both bare and CMC–nZVI suspensions, prepared in NaHCO_3_ electrolyte solution, was quantitatively determined by time-resolved optical absorbance methodology. The change in optical absorbance of nZVI at a wavelength of 508 nm was monitored over time, in suspensions maintained under quiescent conditions, using a UV/VIS Spectrophotometer (GENESYS 10S, Thermo, Madison, WI, USA). The optical absorbance was measured every 3 min for 3 h; all measurements were made at room temperature. All measurements were replicated three times and average values were recorded along with the associated standard deviation.

### 2.4. DLVO Calculations

The classical DLVO theory was used to calculate the total interaction energy, determined as the sum of van der Waals (VDW) attractive and electric double layer (EDL) repulsive forces that exist between nZVI particles. The interaction between two nZVI particles was considered to be a sphere–sphere interaction. Calculation of the EDL repulsion interaction was carried out according to Bhattacharjee and Elimelech [27] as:

(1) $$\Phi_{EDL\left(nZVI-nZVI\right)}=\frac{2\pi a_{p1}a_{p2}n_{\infty }K_{B}T}{\left(a_{p1}+a_{p2}\right)k^{2}}\left[2\psi_{p1}\psi_{p2}ln\left(\frac{1+e^{-ky}}{1-e^{-ky}}\right)+\left(\psi_{p1}^{2}+\psi_{p2}^{2}\right)ln\left(1-e^{-2ky}\right)\right]$$

where $\Phi_{EDL\left(nZVI-nZVI\right)}$ is the EDL interaction energy ($K_{B}T$) between two nZVI particles; $a_{p1}$ and $a_{p2}$, are the radii of the first and second interaction nZVI particles (nm), respectively; $n_{\infty }$, is the bulk number density of ions (-);$\;K_{B}$, is the Bolzmann constant (J K^−1^); T, is the absolute temperature (ͦ K); k, is the inverse Debye–Huckel length (m); $\psi_{p1}$ and $\psi_{p2}$, is the surface potential of the interacting nZVI particles (mV); e, is the electron charge (Coulomb); and y, is the separation distance between two nZVI particles (nm).

The VDW attraction interaction energies for nZVI–nZVI nanoparticles was calculated according to Gregory [28] as:

(2) $$\Phi_{VDW\left(nZVI-nZVI\right)}=-\frac{A_{121}a_{p1}a_{p2}}{6y\left(a_{p1}+a_{p2}\right)}\left[1-\frac{5.32y}{\lambda }ln\left(1+\frac{\lambda }{5.32y}\right)\right]$$

where $\Phi_{VDW\left(nZVI-nZVI\right)}$ is the VDW attraction interaction energy ($K_{B}T$) between two nZVI particles; $A_{121}$, is the Hamaker constant (J); and λ is the characteristics wavelength of the interaction (nm).

## 3. Results

### 3.1. Characteristics of the Synthesized nZVI

**Figure 1:**
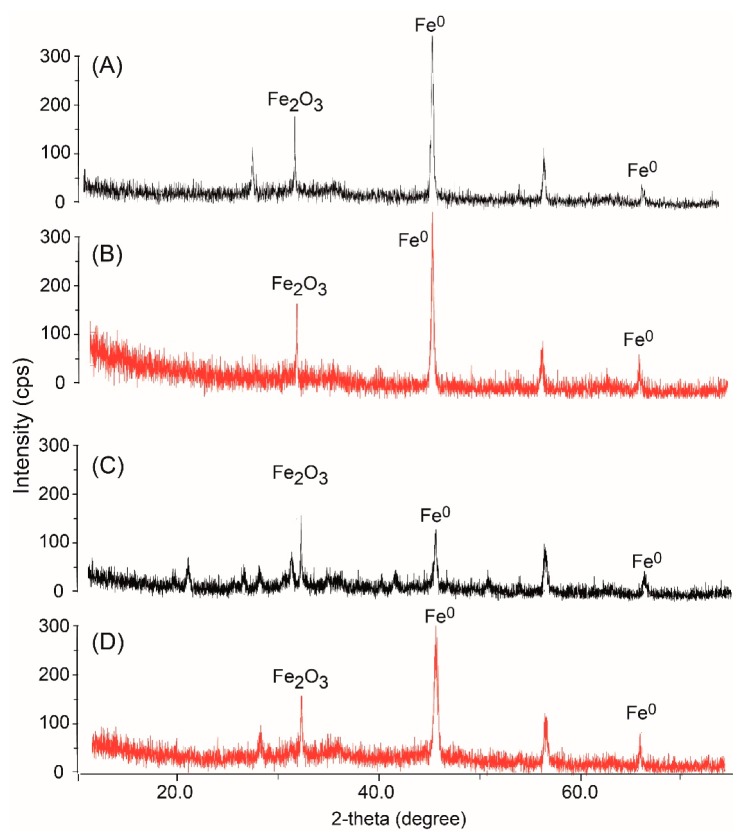
X-ray diffraction (XRD) patterns of (**A**) bare nanoscale zero-valent iron (nZVI), and (**B**) carboxymethyl cellulose (CMC)–nZVI at time of preparation; and of (**C**) bare nZVI and (**D**) CMC–nZVI, aged for 30 days.

The crystalline structure of the synthesized nZVI as determined by XRD patterns revealed that Fe^0^ was the dominant form in the prepared iron powder. This was confirmed by the characteristic diffraction peaks 2θ = 45.05° and 65.93°; and 2θ = 44.84° and 65.64° (JCPDS No. 06-0696) for the bare and CMC–nZVI, respectively (Figure 1A,B) [29]. Peaks of iron oxide were also present in the prepared iron powder (2θ = 31.34° and 31.1° for the bare and CMC–nZVI, respectively). XRD analysis was repeated after one month of preparation to examine the effect of aging on the structure of nZVI particles. Diffraction peaks at 2θ around 45° and 65° of Fe^0^ remained visible in the crystalline patterns in both bare and CMC–nZVI (Figure 1C,D). However, the intensity of the Fe^0^ peaks were significantly decreased in bare nZVI after an aging period of 30 d. In contrast, the intensity of the Fe^0^ peaks of the CMC–nZVI only slightly decreased as a result to the aging period. These results indicate that the CMC polymer stabilizer reduced the degree of oxidation of Fe^0^ to iron oxides in the prepared nZVI after an aging period of 30 d. Previous research also showed that the presence of CMC coating prevented the oxidation of Fe^0^ after 30 days of preparation [30].

**Figure 2:**
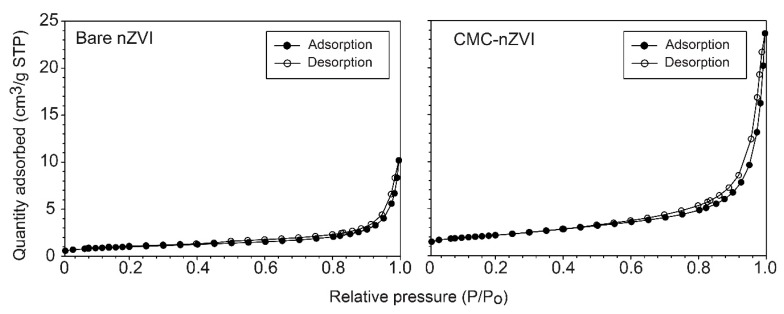
Effect of CMC polymer stabilizer on the Brunauer, Emmett and Teller (BET) adsorption/desorption isotherms of nZVI particles.

In the presence of CMC polymer stabilizer, the total surface area was increased from 3.55 ± 0.02 m^2^ g^−1^ for bare nZVI to 7.61 ± 0.01 m^2^ g^−1^ for the CMC–nZVI. The increase in surface area between coated CMC–nZVI and bare nZVI is caused by the polymer coating of the particles, which prevents agglomeration [31]. CMC–nZVI also showed larger pore volume and smaller pore size. Barrett-Joyner-Halenda (BJH) desorption average pore volume was increased from 0.017 to 0.034 cm^3^ g^−1^, and BJH desorption average pore size was reduced from 191.1 to 178.7 Å, for bare and CMC–nZVI, respectively. The calculated pore size indicated the presence of mesopores in both bare and CMC–nZVI particles. The maximum quantity adsorbed of the N_2_ gas was increased from 10.6 to 23.5 cm^3^ g^−1^ STP for the bare and CMC–nZVI nanoparticles, respectively. Adsorption/desorption isotherms of both bare and CMC–nZVI were type II adsorption isotherms, and showed a very narrow hysteresis loop that fully closed at relative pressure of 0.45 (Figure 2).

**Figure 3:**
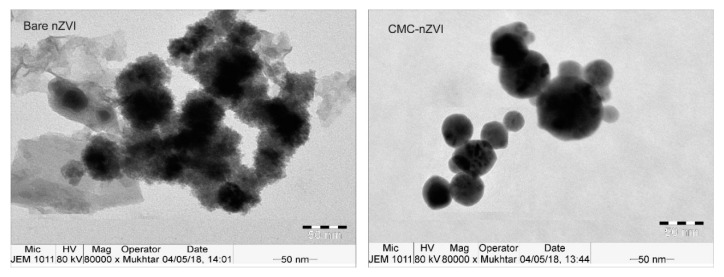
Transmission electron microscope (TEM) images of bare and CMC–nZVI particles.

TEM images showed that CMC–nZVI particles were more spherical in shape with an average diameter in the range of 20–50 nm. Bare nZVI particles showed larger average diameter in the range of 30–70 nm, and more condensed chain-like structure due to increased magnetic interactions and aggregation of particles (Figure 3) [32].

### 3.2. Hydrodynamic Size of nZVI

As nZVI particles are suspended in an electrolyte solution, they tend to agglomerate, forming aggregates of different size depending on particle concentration and chemical conditions in the suspension. Dynamic Light Scattering (DLS) considers the size of any particle (or aggregate of particles) with certain equivalent diameter to be similar in size. Therefore, size average diameters determined by DLS cannot be directly compared to TEM diameters [33]. The hydrodynamic size of nZVI measured by DLS was in general larger than the size range measured by TEM. At nZVI particle concentration of 0.1 g L^−1^, the hydrodynamic size of nZVI ranged between 107.4–188.1 and 92.6–145.3 nm for bare and CMC–nZVI, respectively (Table 1). The hydrodynamic size of CMC–nZVI nanoparticles was smaller than that of bare nZVI nanoparticles at all experimental pH and IS values. However, the effect of the CMC coating on the average reduction in the hydrodynamic size of nZVI particles was more pronounced at lower IS values, and reached 14.1, 12.1, and 7.8% of the hydrodynamic size of bare nZVI nanoparticles at 1, 50, and 100 mM, respectively.

At nZVI particle concentration of 1.0 g L^−1^, much larger hydrodynamic sizes for nZVI nanoparticles were observed at all pH and IS values. The hydrodynamic size of the nZVI nanoparticle ranged between 160.2–1939.1 and 132.7–267.3 nm for the bare and CMC–nZVI nanoparticles, respectively (Table 1). Increasing particle concentration likely increased aggregation and resulted in greater settling profile. This is mainly attributed to the higher probability that two particles will collide to form an aggregate as the number of particles increase due to higher concentration [15]. In contrast to previous results with the lower 0.1 g L^−1^ particle concentration, the effect of the CMC coating on the average reduction of the hydrodynamic size of the nZVI nanoparticles at particle concentration of 1.0 g L^−1^ was more pronounced at larger IS values, and reached 21.7, 67.2, and 63.5% of the hydrodynamic size of bare nZVI nanoparticles at 1, 50, and 100 mM, respectively (Table 1).

### 3.3. Zeta Potential of nZVI

**Figure 4:**
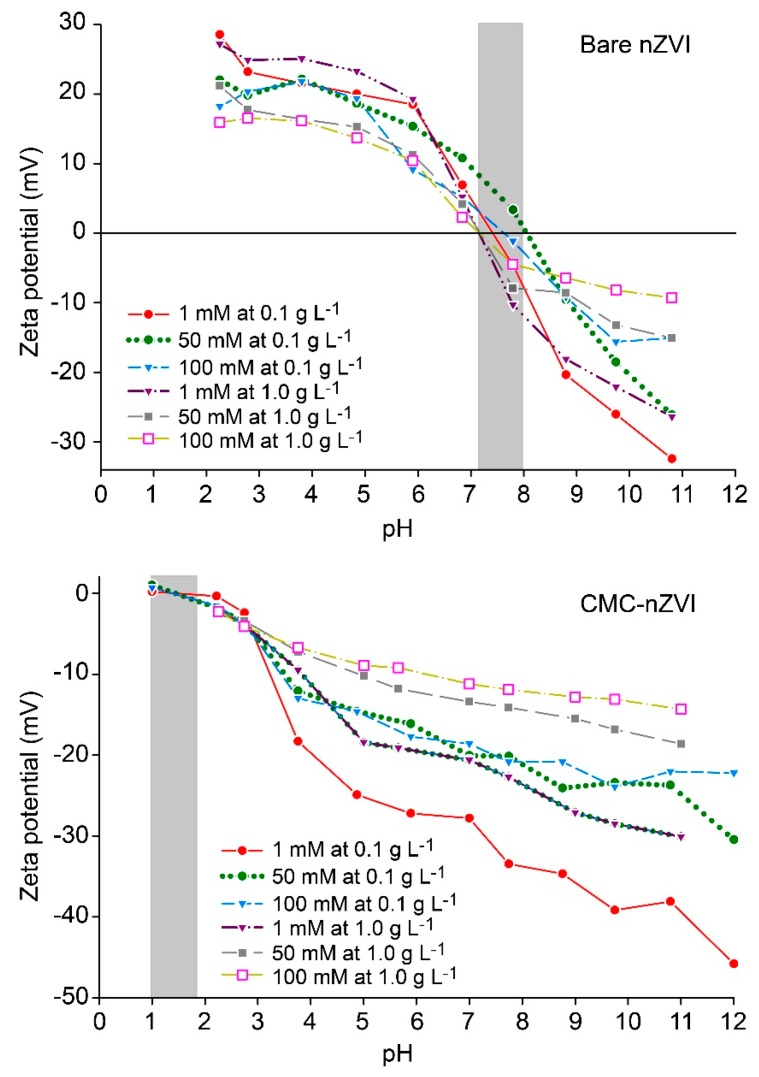
Effect of CMC polymer stabilizer, pH, and IS on the zeta potential of nZVI particles.

Zeta potential of the synthesized nZVI was greatly affected by the chemical conditions of nZVI suspensions. For bare nZVI, zeta potential was always positive at low pH (<7.2–7.9) and negative at high pH (>7.2–7.9). The average PZC of bare nZVI was at pH 7.6 and 7.3 for the 0.1 and 1.0 g L^−1^ nZVI particle concentration, respectively (Figure 4). Increasing the IS from 1 to 100 mM resulted in a decrease in zeta potential of nZVI. This is mainly attributed to the shrinkage of the extent of the electrical double layer at high IS, and the subsequent decrease in the electrostatic repulsion forces [34]. For example, at pH 11, zeta potential of bare nZVI was –32.4, –26.1, and –15.1 mV (at nZVI particle concentration 0.1 g L^−1^), and –26.4, –15.3 and –9.3 mV (at nZVI particle concentration 1.0 g L^−1^) for IS 1, 50, and 100 mM, respectively (Table 2).

For CMC–nZVI, zeta potential was always negative at all measured pH values (measurement range was 2 to 12). The average PZC for the CMC–nZVI was at pH 1–2 (Figure 4). Zeta potential of CMC–nZVI was reduced by the increase of IS and nZVI particle concentration. For example, at pH 11, the zeta potential of CMC–nZVI nanoparticles was –38.1, –27.8 and –22.1 mV (at nZVI particle concentration 0.1 g L^−1^), and –30.1, –18.6 and –14.3 mV (at nZVI particle concentration 1.0 g L^−1^) for IS increased 1, 50, and 100 mM, respectively (Table 2). At pH value greater than 5, stabilizing nZVI by CMC coating resulted in an increase in the zeta potential of the CMC–nZVI as compared to that of the bare nZVI at all IS and particle concentrations. The extent of the increase in the zeta potential ranged between one- to four-fold, and was greater at pH values close to PZC (7.3–7.6), high IS, and larger particle concentration (i.e., conditions favoring aggregation) (Table 2). At larger zeta potential, the extent of the electrostatic interactions between nZVI particles increased leading to more stable nZVI [33].

### 3.4. Effect of pH on the Stability of nZVI

**Figure 5:**
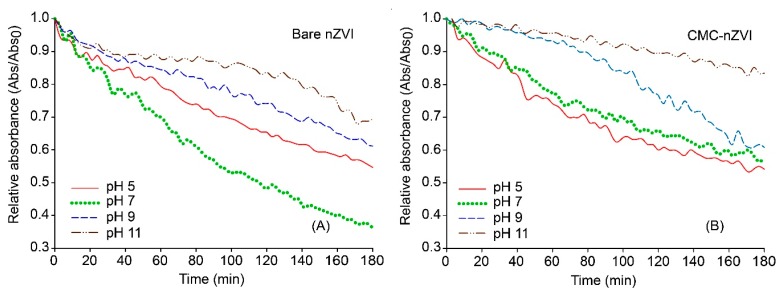
Effect of pH on the stability bare nZVI, (A); and CMC–nZVI, (B), of 0.1 g L^−1^ nZVI suspensions in 1 mM NaHCO_3_.

Sedimentation tests showed the occurrence of a decrease in absorbance over time. This was mainly attributed to the reduction in the number of particles in the suspension due to aggregation, as well as the settlement of heavier aggregates out of solution over time. The stability of nZVI was highly determined by the hydrodynamic size and zeta potential of the particles. Factors favoring smaller particle size and larger zeta potential enhanced nZVI stability. Relative absorbance of bare nZVI after 3 h was 0.69, 0.61, 0.54, and 0.36 at pH values of 11, 9, 5, and 7, respectively (Figure 5), which is consistent with the decrease in the values of zeta potential.

The same trend was also observed for CMC–nZVI, where relative absorbance after 3 h was 0.83, 0.62, 0.57, and 0.55 at pH values of 11, 9, 7, and 5, respectively (Figure 5). Under our experimental conditions, CMC polymer stabilizer greatly increased the stability of nZVI particles. For example, at low IS (1 mM) and pH 7, dynamic stability was lowest with bare nZVI (pH of the PZC was 7.4) and relative absorbance was reduced to 0.36 after 3 h. Under the same chemical conditions but in the presence of CMC coating, relative absorbance of CMC–nZVI was reduced to only 0.57 after 3 h (Figure 5). These results indicate an enhancement in the stability of CMC–nZVI nanoparticles by 58.3% as compared to that of bare nZVI nanoparticles under the aforementioned solution chemistry conditions. Similar findings were reported by Cirtiu et al. [31] who evaluated the stability of nZVI nanoparticles in aqueous NaHCO_3_, and reported similar enhancement in the stability of nZVI with several polymer-coating materials.

**Figure 6:**
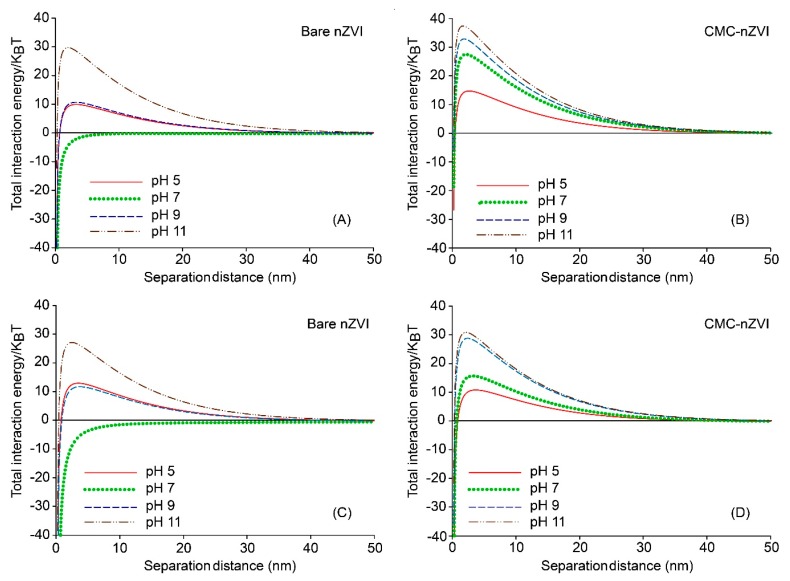
Effect of pH and CMC polymer stabilizer on the total interaction energy of 0.1 g L^−1^ (**A**,**B**) and 1.0 g L^−1^ (**C**,**D**) nZVI particle suspensions in 1 mM NaHCO_3_.

Calculations of the DLVO total interaction energy between nZVI particles were in agreement with stability results. At low IS (1 mM), substantial energy barriers between nZVI particles were formed indicating unfavorable attachment conditions (i.e., domination of electrostatic repulsion forces). The same behavior was observed for both the 0.1 g L^−1^ (Figure 6A,B) and 1.0 g L^−1^ (Figure 6C,D) nZVI particle concentration. The energy barrier was slightly larger in the presence of CMC and at the lower nZVI particle concentration. The only exception was with bare nZVI at pH 7 where the total interaction was dominated by van der Waals attraction forces. It is worth mentioning that, for bare nZVI at IS of 1 mM and pH 7, zeta potential was at its minimum (6.9 and 5.2 mV) (Table 2), and the hydrodynamic size of bare nZVI was at its maximum (132.1 and 287.2 nm) (Table 1) for the nZVI particle concentrations of 0.1 and 1.0 g L^−1^, respectively. In other words, conditions were totally in favor of particle aggregation and therefore no energy barrier was observed at pH 7 as compared to all other pH values, and nZVI particles were irreversibly aggregated in the primary energy minimum as a result to the domination of attraction forces.

The maximum energy barrier occurred at pH 11 and reached 29.7 and 37.4 $K_{B}T$ for the 0.1 g L^−1^ bare and CMC–nZVI, respectively. For the 1.0 g L^−1^ bare and CMC–nZVI, the maximum energy barrier was slightly less and reached 27.1 and 30.9 $K_{B}T$, respectively (Figure 6). In general, the energy barrier decreased with decreasing pH for the 0.1 g L^−1^ CMC–nZVI nanoparticles and reached 32.8, 27.5, and 14.7 $K_{B}T$ at pH 9, 7, and 5, respectively. For the 0.1 g L^−1^ bare nZVI nanoparticles, the energy barrier decreased with decreasing pH and reached 10.7 and 9.9 $K_{B}T$ at pH 9 and 5, respectively. The decrease in the energy barrier with decreasing pH was slightly larger for the 1.0 g L^−1^ CMC–nZVI nanoparticles and reached 28.8, 15.7, and 10.8 $K_{B}T$ at pH 9, 7, and 5, respectively. For the 1.0 g L^−1^ bare nZVI nanoparticles, the energy barrier decreased at pH 9 and 5 to 11.7 and 12.9 $K_{B}T$, respectively (Figure 6).

### 3.5. Effect of IS on the Stability of nZVI

**Figure 7:**
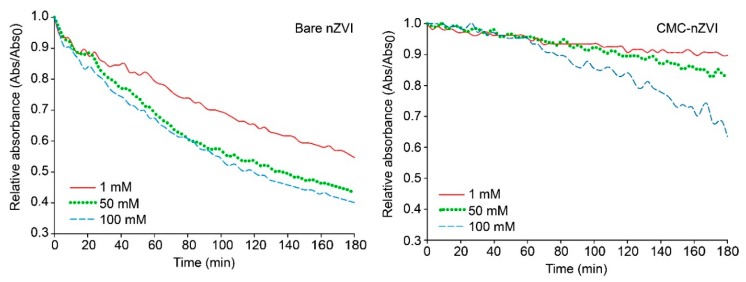
Effect of CMC polymer stabilizer and IS on the stability of 0.1 g L^−1^ nZVI particle suspensions at pH 9.

Increasing IS of the background electrolyte solution resulted in a decrease in the stability of nZVI nanoparticles regardless of the concentration of the nZVI particles. Figure 7 shows the effect of increasing IS on dynamic stability of 0.1 g L^−1^ bare and CMC–nZVI at pH 9. Within 1 h, relative absorbance of bare nZVI was reduced to 0.82, 0.71, and 0.65 at IS values of 1, 50, and 100 mM, respectively. In contrast, CMC polymer stabilizer enhanced the stability of nZVI, and relative absorbance of CMC–nZVI after 1 h remained above 0.95 at all IS values. Substantial decrease in the relative absorbance of bare nZVI occurred after 3 h, and relative absorbance reached 0.55, 0.43, and 0.4 at IS values of 1, 50, and 100 mM, respectively. Even after 3 h of sedimentation, most of the CMC–nZVI remained stable, and relative absorbance was 0.89, 0.83, and 0.63 at IS values of 1, 50, and 100 mM, respectively (Figure 7). These findings indicate that CMC polymer coating increased the dynamic stability of nZVI by 61.8, 93.1, and 57.5% as compared to that of bare nZVI at IS values of 1, 50, and 100 mM, respectively. It is interesting to mention that the largest increase in the dynamic stability of nZVI due to the presence of CMC polymer coating was observed at IS between 1–50 mM (93.1%). At low IS (≤1 mM), electrostatic repulsive forces will dominate and most nZVI suspensions will have greater stability, therefore, the impact of CMC will be limited at this IS. On the other hand, at high IS (≥50–100 mM) attractive forces will dominate and most nZVI particles will settle down even in the presence of stabilizing materials.

**Figure 8:**
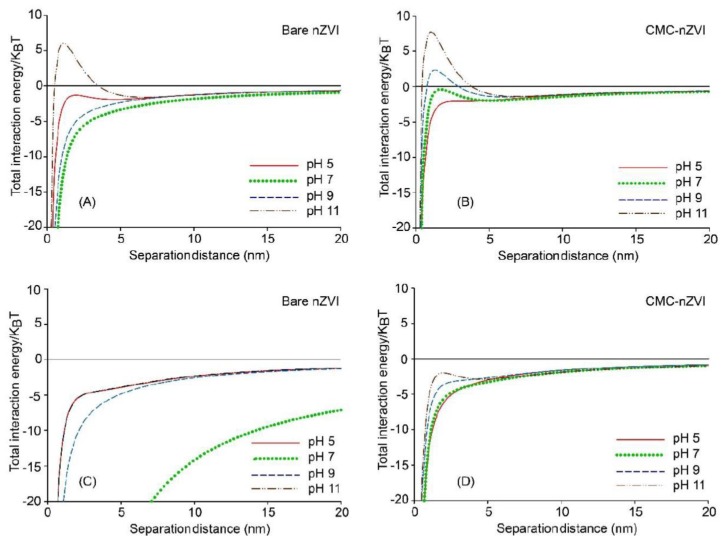
Effect of pH and CMC polymer stabilizer on the total interaction energy of 0.1 g L^−1^ (**A**,**B**) and 1.0 g L^−1^ (**C**,**D**) nZVI particle suspensions in 50 mM NaHCO_3_.

**Figure 9:**
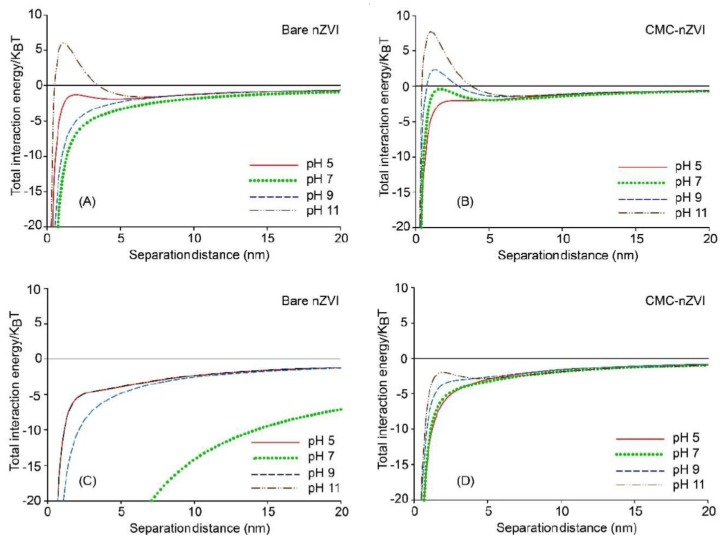
Effect of pH and CMC polymer stabilizer on the total interaction energy of 0.1 g L^−1^ (**A**,**B**) and 1.0 g L^−1^ (**C**,**D**) nZVI particle suspensions in 100 mM NaHCO_3_.

The effect of IS on the dynamic stability of nZVI was also supported by the results of the DLVO calculations. Figure 8 and Figure 9 show the total interaction energy of 50 and 100 mM NaHCO_3_ suspensions of both bare and CMC–nZVI at variable pH (5–11) and particle concentration (0.1 and 1.0 g L^−1^). At IS 50 and 100 mM, interaction energy at almost all pH and particle concentrations was dominated by attractive forces, favoring irreversible aggregation of the nZVI particles in the primary energy minimum. Only two exceptions were observed. The first was with bare nZVI at IS 50 mM, 0.1 g L^−1^ particle concentration, and pH 11, where a small energy barrier was formed around 6 $K_{B}T$. The second exception was with the CMC–nZVI at IS 50 mM, 0.1 g L^−1^ particle concentration, and pH 9 and 11, where small energy barriers were formed around 2 and 7 $K_{B}T$, respectively (Figure 8A,B). According to the DLVO theory, energy barriers >20 $K_{B}T$ are required for particles to remain stable in suspensions for long times. Therefore, the small energy barriers that were formed with these exceptions will easily vanish as the separation distance between two nZVI particles decreases, and the extent of the van der Waals attractive forces will increase, leading to aggregation of nZVI particles. 

Our results suggest that at IS above 50 mM and nZVI particle concentration larger than 0.1 g L^−1^ the likelihood of nZVI aggregation is high. Nevertheless, polymer stabilizer would enhance the stability of nZVI even under these unfavorable solution chemistry. This is clearly visible in Figure 8 and Figure 9 where we can see that even for nZVI suspensions dominated by attractive forces, the extent of the attractive forces is less when nZVI particles are coated with CMC.

## 4. Conclusions

In this research, we investigated the effect of pH, IS, nZVI particle concentration, and CMC polymer stabilizer on the stability and dynamic aggregation of nZVI suspensions. Increased surface area and smaller pore size were observed in the presence of CMC polymer stabilizer. XRD patterns revealed a decrease in Fe^0^ peaks over time with bare nZVI, whereas Fe^0^ in CMC–nZVI remained without significant change for 30 d. The hydrodynamic size of nZVI was always smaller in the presence of CMC, particularly at larger IS and nZVI particle concentrations. The average PZC for bare and CMC–nZVI was at pH 7.3–7.6, and 1–2, respectively. The increase in the zeta potential of CMC–nZVI reached one- to four-fold of that for bare nZVI, and was greater at pH values close to PZC, high IS, and larger particle concentration. CMC increased nZVI stability and relative absorbance remained above 0.95 after 1 h of sedimentation, while it was reduced to 0.65 with bare nZVI. Our results suggest that at IS above 50 mM and nZVI particle concentration larger than 0.1 g L^−1^, the likelihood of nZVI aggregation is high. Under these conditions, maximum particle aggregation is expected, therefore, it is extremely important to use polymer stabilizer to enhance the stability and transport of nZVI particles, and subsequently increase their efficiency in environmental applications.

## Figures and Tables

**Table 1:** Effect of nZVI particle concentration, CMC polymer stabilizer, ionic strength (IS) and pH on the hydrodynamic size of nZVI.

IS *	pH	nZVI Particle Concentration			
		0.1 g L^−1^		1.0 g L^−1^	
		Bare nZVI	CMC–nZVI	Bare nZVI	CMC–nZVI
1 mM	5	115.2 ± 3.9	100.7 ± 3.2	172.1 ± 53.2	164.1 ± 2.8
	7	132.1 ± 5.9	116.2 ± 5.3	287.2 ± 25.8	174.9 ± 19.2
	9	122.4 ± 9.7	101 ± 4.2	186.3 ± 18.4	159.6 ± 7.4
	11	107.4 ± 5.1	92.6 ± 6.6	160.2 ± 7.1	132.7 ± 12.2
50 mM	5	130.2 ± 6.4	126.1 ± 7.6	244.1 ± 19.2	166.4 ± 1.7
	7	188.1 ± 12.6	145.3 ± 20.9	1462.8 ± 79.2	193.3 ± 5.4
	9	128.5 ± 6.9	117.7 ± 6.1	263.5 ± 38.7	166.4 ± 2.9
	11	136.2 ± 2.3	123.4 ± 12.2	239.4 ± 13.2	197.6 ± 4.3
100 mM	5	148.3 ± 7.9	137.4 ± 3.8	298.8 ± 32.4	261.2 ± 2.5
	7	172.4 ± 9.0	150.3 ± 2.9	1939.1 ± 234.2	267.3 ± 21.2
	9	149.2 ± 4.8	140.8 ± 5.7	278.1 ± 24.3	241.3 ± 15.7
	11	151.7 ± 18.4	144.7 ± 7.2	286.2 ± 32.1	252.4 ± 14.2

* IS, ionic strength (NaHCO_3_ was used as the background electrolyte solution), numbers are presented followed by the standard deviation (±1 SD).

**Table 2:** Effect of nZVI particle concentration, CMC polymer stabilizer, IS and pH on zeta potential (mV) of nZVI.

IS *	pH	nZVI Particle Concentration			
		0.1 g L^−1^		1.0 g L^−1^	
		Bare nZVI	CMC−nZVI	Bare nZVI	CMC−nZVI
1 mM	5	20.3	−24.9	19.3	−18.4
	7	6.9	−30.3	5.2	−20.6
	9	−20.4	−34.7	−18.1	−27.1
	11	−32.4	−38.1	−26.4	−30.1
50 mM	5	18.6	−16.7	15.3	−10.2
	7	10.7	−20.1	4.2	−13.3
	9	−9.5	−23.4	−8.6	−15.5
	11	−26.1	−27.8	−15.1	−18.6
100 mM	5	19.4	−18.2	13.7	−8.9
	7	4.2	−18.9	2.3	−11.2
	9	−9.2	−20.8	−5.1	−12.8
	11	−15.1	−22.1	−9.3	−14.3

* IS, ionic strength (NaHCO_3_ was used as the background electrolyte solution).
